# Inaccurate DNA Synthesis in Cell Extracts of Yeast Producing Active Human DNA Polymerase Iota

**DOI:** 10.1371/journal.pone.0016612

**Published:** 2011-01-31

**Authors:** Alena V. Makarova, Corinn Grabow, Leonid V. Gening, Vyacheslav Z. Tarantul, Tahir H. Tahirov, Tadayoshi Bessho, Youri I. Pavlov

**Affiliations:** 1 Institute of Molecular Genetics of Russian Academy of Science, Moscow, Russian Federation; 2 Eppley Institute for Research in Cancer, University of Nebraska Medical Center, Omaha, Nebraska, United States of America; National Cancer Institute, United States of America

## Abstract

Mammalian Pol ι has an unusual combination of properties: it is stimulated by Mn^2+^ ions, can bypass some DNA lesions and misincorporates “G” opposite template “T” more frequently than incorporates the correct “A.” We recently proposed a method of detection of Pol ι activity in animal cell extracts, based on primer extension opposite the template T with a high concentration of only two nucleotides, dGTP and dATP (incorporation of “G” versus “A” method of Gening, abbreviated as “misGvA”). We provide unambiguous proof of the “misGvA” approach concept and extend the applicability of the method for the studies of variants of Pol ι in the yeast model system with different cation cofactors. We produced human Pol ι in baker's yeast, which do not have a *POLI* ortholog. The “misGvA” activity is absent in cell extracts containing an empty vector, or producing catalytically dead Pol ι, or Pol ι lacking exon 2, but is robust in the strain producing wild-type Pol ι or its catalytic core, or protein with the active center L62I mutant. The signature pattern of primer extension products resulting from inaccurate DNA synthesis by extracts of cells producing either Pol ι or human Pol η is different. The DNA sequence of the template is critical for the detection of the infidelity of DNA synthesis attributed to DNA Pol ι. The primer/template and composition of the exogenous DNA precursor pool can be adapted to monitor replication fidelity in cell extracts expressing various error-prone Pols or mutator variants of accurate Pols. Finally, we demonstrate that the mutation rates in yeast strains producing human DNA Pols ι and η are not elevated over the control strain, despite highly inaccurate DNA synthesis by their extracts.

## Introduction

Cancer etiology is clearly connected to a cell's ability to remove or tolerate lesions in DNA by repair and translesion DNA synthesis. DNA polymerases of the Y family are involved in both of these processes and play a pivotal role in the prevention of cancer, as exemplified by the rapid accumulation of skin tumors in patients with xeroderma pigmentosum variant (XP-V) syndrome lacking Pol η [1]; [2].

**Figure 1:**
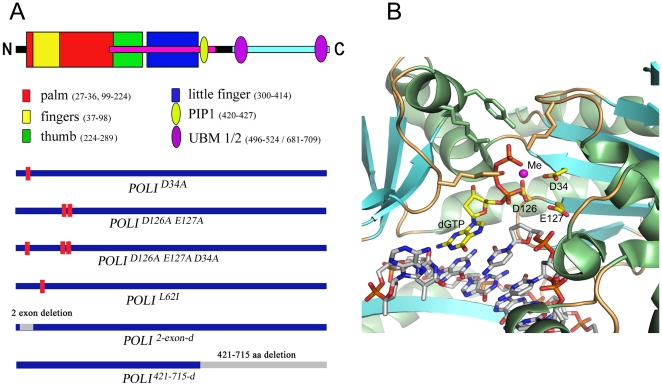
Structure of Pol ι and the variants studied. A. Upper half. The schematic domain structure of Pol ι is shown (PIP is the protein interaction domain, UBM – ubiqutin-binding motif). Lower half. The Pol ι mutant variants were used in the study (Red bars on the thick blue line representing Pol ι show the positions of amino acid changes in polymorphic variants of Pol ι. Grey bars indicate deletions for truncated Pol ι variants): *hPOLISc^2exon-d^* (Pol ι ^2exon-d^) – Pol ι variant with a deletion of exon 2 (encoding for amino acids 14-55) representing an alternative splice variant of human and mouse Pol ι; *hPOLISc^D34A^* (Pol ι ^D34A^) and *hPOLISc^D126A/E127A^* (Pol ι ^D126A/E127A^) – “catalytically dead” Pol ι variants created as amino acid substitutions of evolutionary conservative Asp34 or Asp126 and Glu127 to Ala; *hPOLISc^D34A/D126A/E127A^* (Pol ι^ D34A/D126A/E127A^) – a triple “catalytically dead” Pol ι variant with amino acid substitutions of Asp34, Asp126 and Glu127 to Ala; *hPOLISc^L62I^* (Pol ι^L62I^) – Pol ι variant with a substitution of evolutionary polymorphic amino acid Leu62 to Ile; *hPOLISc^42I-612-d^* variant with a deletion of the C-terminal half of Pol ι is shown to illustrate what minimal part retains enzymatic activity and whose crystal structure has been determined. B. Polι active site. A close view at the Pol ι active site in ternary complex with DNA (template T) and incoming dGTP (3gv8). Pol ι and DNA molecules are represented as cartoon and sticks, respectively. The incorporated nucleotides and active site residues Asp34, Asp126 and Glu127 are represented by sticks and highlighted with yellow carbons. The side chains of phosphate-binding residues are also shown as sticks. The metal ion is drawn as a magenta ball.

In mammals, a close relative of Pol η, DNA polymerase iota [3] (Pol ι, Fig. 1A) is unique because it violates the Watson-Crick rules of DNA synthesis, incorporating “G” opposite template “T” more frequently than the correct “A” [4]–[6] due to the special organization of the active site [7]; [8] (Fig. 1B). It also was shown to possess dRP lyase activity [9]; [10]. In addition, Pol ι is much more efficient in the presence of Mn^2+^ in comparison to Mg^2+^ [6]. It is still unknown what exact biological processes utilize the unusual biochemical properties of Pol ι. It has been proposed that Pol ι participates in immunoglobulin somatic hypermutation (SHM) [11]; [12], bypass of deaminated cytosines [13], several adducts of the purine bases [14]; [15], DNA strand crosslinks [16] and is involved in DNA repair under oxidative stress [17]. The role of Pol ι in cancer is two-sided [18]. Pol ι levels are elevated in some tumors and tumor cells [19]–[23]. In the cells from XP-V patients lacking Pol η, Pol ι is responsible for the high frequency of UV-induced mutagenesis, and ultimately malignant transformation [24]. Defects and polymorphisms in the *POLI* are associated with an increased risk of lung cancer [25]; [26] and 129/J mice, devoid of Pol ι, are prone to an elevated occurrence of UV-induced skin tumors [27]; [28].

The number of model organisms or cell lines with defects of the gene encoding for Pol ι, which are instrumental to understanding the function of this DNA Pol, is limited. The common mouse strain 129/J, widely used as a source of somatic and embryonic cells for cloning, appeared to be Pol ι null due to the presence of a stop-codon in exon 2 [29]. Human Burkitt's lymphoma cell lines with no detectable Pol ι were created by gene targeting by replacement/interruption of exons 1–3 [12]. These two models provided controversial answers to the question of the involvement of Pol ι in SHM. At the present time, it is unclear if these discrepancies imply that one of the “knockout” model systems is somehow flawed [30] or the mechanisms of SHM in mice and humans or *in vivo versus* Burkitt's lymphoma cell lines are different. Functional analysis of Pol ι variants resulting from deletion and point mutations representing polymorphic *POLI* alleles is required to better understand the role of this Pol.

Preferential misincorporation of G *versus* A opposite the T template is detectable by gel electrophoresis in crude extracts of animal cells (Method of Gening, abbreviated here as misGvA [31]; [32]). In this method, we examined the ability of cell extracts to elongate radio-labeled primer by the competitive incorporation of A or G deoxyribonucleotides opposite template T [32]. Primers of the same length but terminated with A or G slightly differ by their mobility on denaturing polyacrylamide gel. The relative misincorporation of G versus incorporation of A is readily visualized by the intensity of a band with slower mobility in a doublet band. The technique detected activity of Pol ι in several organs of mammals [32]; [33], while no misGvA activity was seen in the same organs in other vertebrates in the presence of Mg^2+^ [34]. Inaccurate DNA synthesis due to Pol ι activity was detected in human melanomas [21]; [35].

The misGvA activity was not detected in most organs of the 129/J strain mice, which is supposed to be Pol ι null. Quite unexpectedly, weak misGvA was found in the extracts of the brain of these mice when Mg^2+^ ions were used as a cofactor of DNA-polymerase reaction [32]. These results could be explained in several ways. One proposal is that there is some mechanism of suppression of the nonsense mutation in the brains of 129/J mice, for example alternative splicing of the exon part with nonsense mutation [30]; [32]. Another explanation is that the misGvA method is not 100% specific for Pol ι and allows for the detection of other inaccurate Pols, for example Pol η.

In the current paper we have used a novel version of the misGvA method with crude extracts of yeast cells and improved the misGvA technique for the specific detection of Pol ι by inclusion of the Mn^2+^ ion in the reaction protocol. We examined the limitations and potential applications of the method and studied the activity of several strategic variants of Pol ι schematically shown in Fig. 1A. The misGvA technology was very useful for the functional analysis of several variants of human Pol ι produced in yeast. We demonstrated the pivotal role of active site D34 residue in Pol ι activity, along with previously studied D126 and E127. We have found that enzyme without the part encoded by exon 2 is inactive and the L62I change, which was proposed to have evolutionary significance [34], has virtually no effect on activity. We have also produced human Pol η in yeast and demonstrated that its low fidelity primer extension can be easily detected in extracts, though the pattern of bands was different from Pol ι. We propose that the method could be adapted to study the fidelity of DNA replication by the extracts of yeast producing various inaccurate DNA Pols. Finally, we have found that the inaccuracy of DNA synthesis in yeast producing human DNA Pols ι and η did not lead to statistically significant elevation of mutation rates in corresponding strains. Apparently, these foreign Pols are excluded from DNA transactions in live yeast cells.

## Results

### misGvA activity in yeast extracts

**Figure 2:**
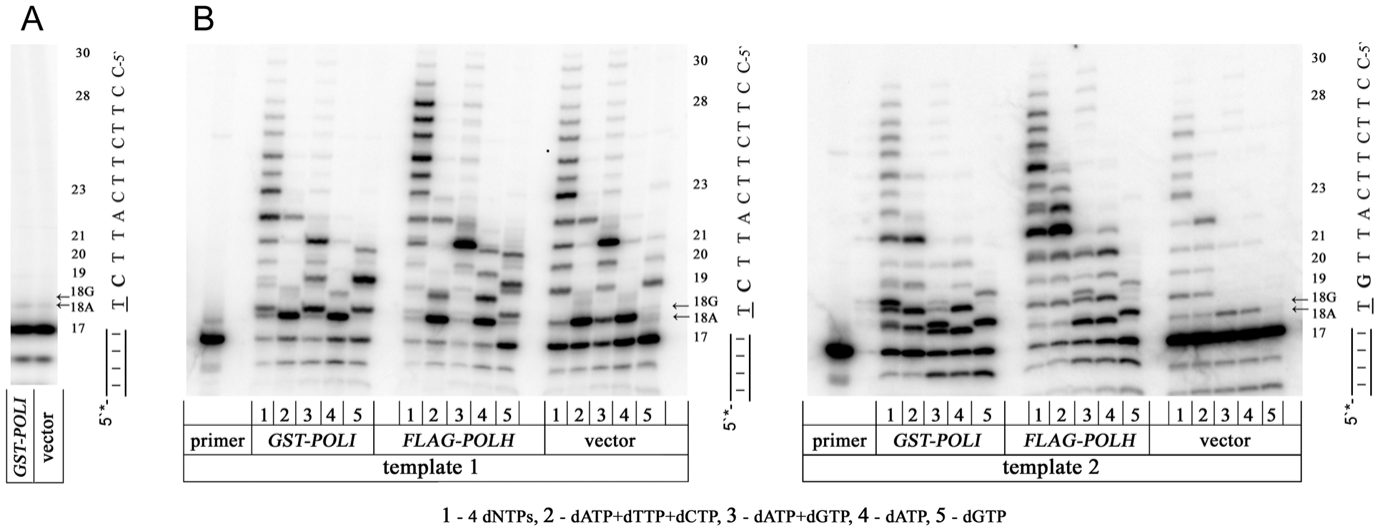
Detection of misGvA activity of Pol ι. A. DNA-polymerase activity of yeast extracts producing Pol ι or transformed by empty vector in the absence of dNTPs in reaction mixture. The reactions were carried out at 37°C for 15 min as described in “Material and Methods” using standard template 1 [4]; [31], with no dNTPs in the reaction mixture. DNA oligonucleotides were separated on 18% polyacrylamide/7 M urea gels and visualized using Storage Phosphor Screen in Typhoon 9700. B. misGvA activity by yeast extracts producing human Pol ι, Pol η or transformed with empty vector. Template 1 (left panel, standard substrate) and template 2 (modified substrate with the substitution of C to G at the position of 19) were incubated with yeast cell extracts and high concentrations of exogenous nucleotides. The activity of Pol ι produced in yeast is detectable by the misincorporation of dGTP opposite template T by whole cell extracts (“misincorporation of G” method of Gening, abbreviated as misGvA). The cells extracts of yeast producing Pol ι, Pol η or containing empty vector were used as an enzymatic preparation for DNA polymerase reaction with P^32^-labeled oligonucleotide substrate in the presence of 0.15 mM Mn^2+^ divalent metal ions and various combinations of equal concentrations of 1 mM dNTPs. For each extract, five different conditions have been used: 1) all dNTPs, 2) dATP, dTTP and dCTP but with dGTP omitted, 3) dGTP and dATP, 4) only dATP, and 5) only dGTP. Template 2 with the substitution of the next nucleotide upstream (+2, corresponding to the position of 19 on elongated primer) after the T template (+1, position of 18) from C to G was used to exclude the possibility of transient misincorporation by the template slippage mechanism. The reactions were carried out at 37°C for 15 min. DNA products were separated on 18% polyacrylamide/7 M urea gels and visualized using Storage Phosphor Screen in Typhoon 9700. The Pol ι activity was determined by the presence of the two “doublet” radioactive bands corresponding to 18-mer oligonucleotides with A or G at the 3′-end, possessing different electrophoretic mobility. The lower band with higher mobility is indicative of the amount of oligonucleotide with 3′-terminal A, whereas the upper band corresponds to the amount of a less mobile oligonucleotide with G incorporated opposite T in position 18 (lines 1 and 3).

We used a variant of the misGvA method for the detection of Pol ι in crude extracts of yeast cells. The reactions included DNA substrates, cell extracts of the strains producing human Pol ι, Mn^2+^ ions and various combinations of deoxynucleotides. Extracts of strains with empty vectors served as a negative control. For each extract, five different conditions have been used: 1) reaction with all four deoxynucleotides, 2) same but with dGTP omitted, 3) with only dGTP and dATP present, 4) with only dATP, and 5) with only dGTP. When none of the exogenous dNTPs were present, the synthesis was extremely weak and same faint bands corresponding to the correct incorporation of dATP at position +1 were detected for the extract of yeast producing Pol ι and the control extract, suggesting that the concentration of dNTPs in the extracts is low and does not support DNA synthesis under our conditions (Fig. 2A). The addition of a high concentration of exogenous dNTPs led to a strong stimulation of DNA synthesis. The misGvA activity was clearly seen for yeast extracts producing Pol ι with all four dNTPs and when dATP and dGTP were present (Fig. 2B, major double band corresponding to 18G and 18A). In these experiments we compared two different oligonucleotide templates and, in addition to a negative control (extract of parent yeast strain transformed by the vector without *POLI* gene), we have used a positive control with extract of the strain producing human Pol η.

The left panel of Fig. 2B represents primer extension reactions with template 1. Under our experimental conditions, crude cell extracts from the control strain with the vector alone possessed almost undetectable exonuclease activity and had moderate DNA polymerase activity with all four dNTPs (right set of reactions in the left panel of Fig. 2B). When dGTP was omitted from the reaction, the synthesis was halted almost exclusively at position 18, likely representing predominant dATP incorporation opposite template T. In the presence of dATP and dGTP, single bands are observed with a major termination site at position 21, when missing dTTP has to be inserted. In addition, the synthesis proceeded as for lane 1 until position 21, because only two nucleotides were required to copy the template. When dATP was present alone, the synthesis was stopped at position 18. The reaction with only dGTP in the absence of dATP was inefficient judging by a higher percent of unutilized primer in line five for the control extract. The band with G at position 19 and minor bands at 18-position could be explained by the better extension of products (versus proofreading) driven by residual dNTPs in cell extracts when dGTP was in great excess. It is also possible that dGTP was incorporated by a template slippage mechanism, when the misaligned template guided incorporation of dGTP opposite +2 “C” in the template and, after immediate realignment. Next, dGTP opposite the same +2 “C” was incorporated [36]; [37]. The dGTP incorporation in the 18 or 19 position did not occur when dATP was present in reactions.

The pattern of band changes drastically when we used the extract from cells producing Pol ι. These extracts possessed elevated DNA polymerase activity, as indicated by the small quantity of non-elongated primer, position 17 (Fig.2B, left set of reactions in the left panel). Apparently, most of this synthesis was due to Pol ι. Most termination falls on the short elongation products, so that the bands at position 18 and 19 are more intense than with control extracts. This is consistent with the low processivity of Pol ι and its inclination to halt replication after the incorporation of nucleotides opposite template T [4]; [8]. In the presence of four dNTPs, the pattern of bands was different from the control. A prominent doublet band at position 18 and a fainter doublet band at position 19 appeared due to misGvA activity. The faster migrating 18-mer corresponds to the correct incorporation of A opposite T and the slower one corresponds to the misincorporation of G, consistent with the preferential incorporation of dGTP opposite T for this template/primer substrate by Pol ι [4]. Two different 19-mers most likely represent elongation of 18A and 18G with dGTP, because the upper band in these doublets disappears when dGTP is excluded from the reaction, but becomes a predominant band when only two nucleotides, dGTP and dATP are present. Lanes 4 and 5 on this panel clearly illustrate that each band in the double band is a result of dATP or dGTP incorporation, respectively.

When we used yeast extract of a strain producing Pol η, a Y-family polymerase occupying the second place in “inaccuracy” ranking [38], the overall synthesis of DNA was even better than with Pol ι (the middle set of the reactions in the left panel of Fig. 2B). With all four nucleotides present, most products were in the range of 24–28 nucleotides. The double band at position 18 was barely detectable with all four nucleotides or with A and G together. Some dGTP misincorporation was seen when only dGTP was supplied and could be explained by a slippage mechanism or by direct misincorporation of dGTP opposite T. It is known that the rate of such events for Pol η is about 40 times lower than for Pol ι [39]. Our reaction time was quite long, however, so the accumulation of the only one possible type of terminal product when only one nucleotide is present is not surprising. Faint 18A product in this lane may be explained in the same way as for control extracts, by effects of the presence of some residual dNTPs in yeast extracts. We concluded that only the Pol ι producing strain possesses robust misGvA activity.

Next, we examined the DNA sequence context on the misGvA activity (right panel of Fig. 2B). We have changed the second template nucleotide from C to G, preventing dGTP incorporation by simple slippage mechanism. The pattern of bands with control yeast extracts resembled the pattern with the previous template, but no misincorporation of dGTP opposite 19C was seen in lane 5, where only dGTP was provided (right set of reactions on right part of Fig. 2B). Extract with Pol ι (left set of reactions on the right panel of Fig. 2B) was very active in the misGvA test and generated the doublet band at position 19 with all four nucleotides, apparently due to a very efficient extension of the 18-mer primer with terminal A. Omission of dGTP eliminated the upper 18G and 19G bands. The best visualization of misGvA activity was seen in the presence of dATP and dGTP – a clear doublet band at position 18 with the same intensity for the 18A and 18G bands. Controls on lanes 4 and 5 were characteristic for Pol ι. The two bands are seen in the presence of dATP, the 18A band and 19A. The “dGTP-T stop” was observed in the reaction with dGTP with no elongation after position G19. The results with extract producing Pol η (central set of reactions in the right panel of Fig. 2B) strongly supported the hypothesis that misGvA activity in this assay is an exclusive property of Pol ι. No double bands were detected at position 18 at lines 1 and 3. Most likely, Pol η produced a faint double band with the previous substrate due to slippage or due to sequence context effects. New double bands seen at position 19 with dGTP and dATP fit the concept of general inaccuracy of Pol η and its ability to use the transient misalignment to generate base pair substitutions. This doublet could be explained by dGTP mis-incorporation opposite G and “apparent” misincorporation of dATP opposite G, because the next two template Ts favor the A incorporation by slippage. Pol η also is able to misincorporate dGTP opposite template G when it forced to do so in the presence of dGTP only, consistent with the known properties of the enzyme [39]. The results of this section confirmed that the activity of Pol ι is readily detected in the whole yeast extracts by the misGvA method.

The production of both human polymerases in yeast did not change the rate of spontaneous forward mutations to canavanine resistance in our tester strain (Table 1). The first three rows in the table summarize the results of the measurement of forward mutation rates in yeast carrying expression vectors (negative control). Mutation rates in strains producing either Pol ι, or Pol η or both of them simultaneously, do not differ from negative controls (next four rows, all confidence limits overlap). As a positive control, we have used transformants with similar construct expressing gene for editing deaminase from lamprey, *pmCDA1* (last row). As expected [40], the mutation rate in this strain was more than ten-fold higher than in the controls.

**Table 1:** Expression of the *hPOLISc* or *hPOLHSc* in yeast is non-mutagenic.

Plasmid in yeast transformants	Rate of forward mutations to canavanine-resistance Can^r^×10^−7^ *
pESC-LEU	4.8
	(1.0–11)
pESC-TRP	4.1
	(2.8–7.5)
pESC-URA	3.6
	(1.7–6.1)
pESC-TRP-*hPOLISc*	10.6
	(3.7–14)
pRS424-GAL-GST-TRP- *hPOLISc*	4.8
	(1.5–8.9)
pESC-URA-*hPOLHSc*	2.7
	(1.3–12)
pESC-URA-*hPOLHSc* and pESC-TRP-*hPOLISc*	4.7
	(2.7–6.9)
pESC-*pmCDA1*	130
	(95–250)

* Median for 12-18 independent cultures. The 95% confidence limits are in parenthesis.

### misGvA activity in extracts of cells producing variants of Pol ι

**Figure 3:**
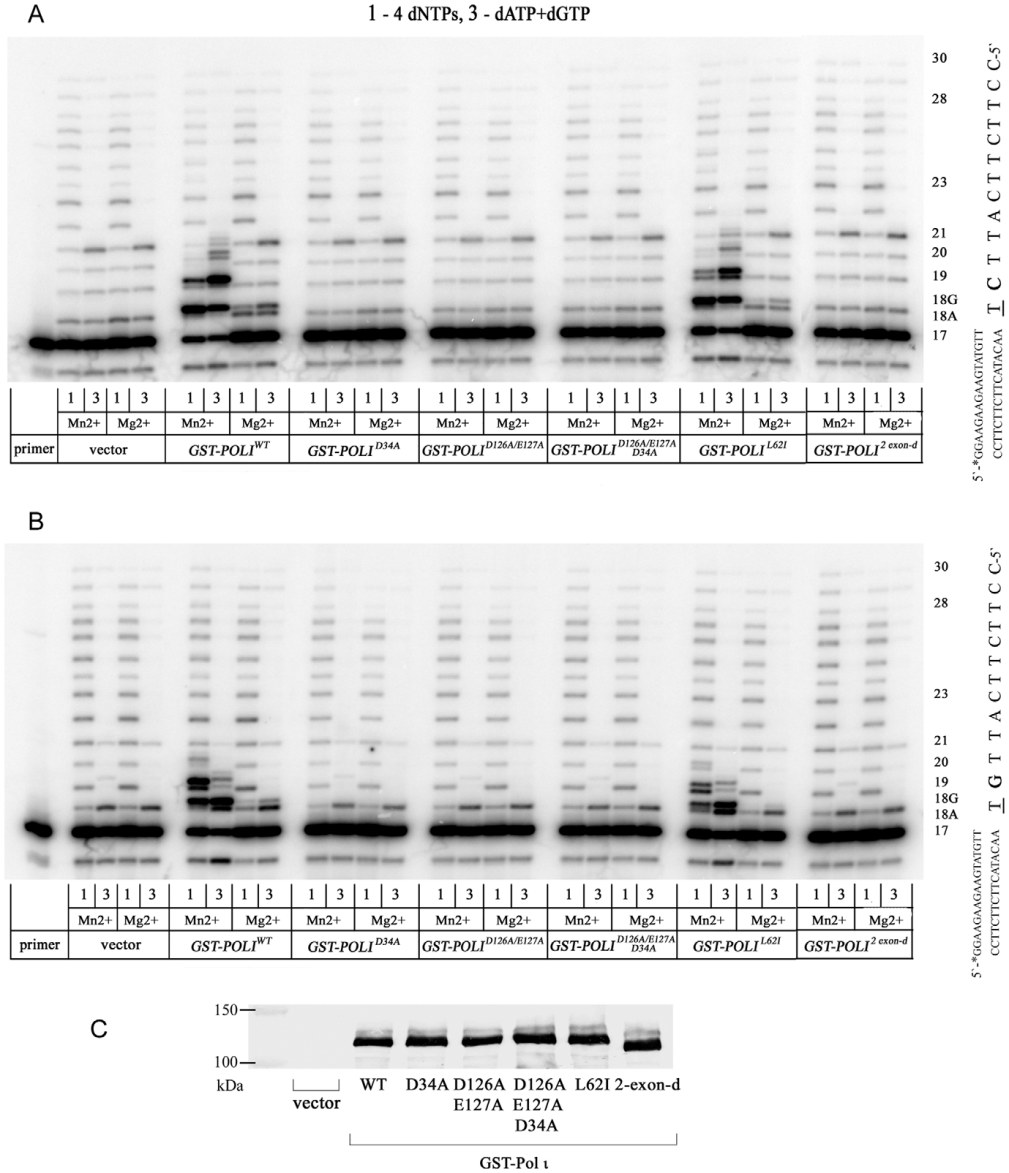
misGvA by yeast extracts producing variants of Pol ι. A. misGvA activity of cell extracts of yeasts producing GST-Pol ι variants was estimated by “misGvA” in 8-min reactions in the presence of 0.15 mM Mn^2+^ and 0.25 mM Mg^2+^ divalent metal ions at template 1 as described in “Materials and Methods.” Two combinations of equal concentrations of 1 mM dNTPs have been used: 1) all dNTPs and 2) dGTP and dATP. B. Same with template 2. B. Western-blot analysis of the production of GST-tagged Pol ι catalytically compromised variants in yeast cells extracts. Extracts containing 40 µg of total protein were separated on 8% polyacrylamide-SDS gel, transferred to a PVDF membrane and probed with polyclonal anti-GST antibodies. Cross-reacting proteins were visualized according to the Western Breeze Chromogenic Anti-Rabbit Kit procedure (Invitrogen).

In the next step, we analyzed the misGvA activity of yeast extracts producing variants of GST-tagged Pol ι in the presence of Mn^2+^ or Mg^2+^ ions (Fig. 3). We have used both template 1 (Fig. 3A) and template 2 (Fig. 3B) to have a possibility of comparing our results with the earlier data with mammalian cell extracts [32]. We performed two types of reactions, with all four nucleotides (lanes labeled “1”) and with dATP and dGTP (lanes labeled “3”). We confirmed the robust production of all Pol ι variants by Western blot with anti-GST antibodies (Fig. 3C). Consistent with the results of the previous section, extracts of cells with vector alone possess some DNA polymerase activity and all bands were consistent with accurate replication. Extracts of cell producing wild-type GST-tagged Pol ι were highly misGvA proficient with Mn^2+^ and were moderately proficient with Mg^2+^ (second set of reactions on the left in Fig. 3AB). The same activity was observed for the Pol ι GST-tagged catalytic core (variant 421–512-d in Fig. 1A.). Pol ι variants with substitutions of conservative amino acids are critical for polymerase reaction according to the crystallographic data (Fig. 1B). In particular, Pol ι^ D34^ with a single amino acid substitution Asp34Ala, Pol ι^ D126A/E127A^ with a double amino acids substitution Asp126Ala, Glu127Ala, and the variant with a triple change were completely inactive. Most of the primer was not extended and only a pattern of bands similar to the control with vector was detected (three central sets of reactions, with either Mn^2+^ or Mg^2+^). We conclude that all three amino acids, D34, D126 and E127 are absolutely necessary for possession of DNA-polymerase activity by human Pol ι. This is consistent with their proposed roles in the coordination of metal ions and the formation of a bond between primer and the incorporating nucleotide based on crystal structures [7]; [8]; [41].

Leu62 is an important amino acid in the active site of Pol ι [8]; [41] and its substitution to Ala decreases Pol ι activity and changes enzyme fidelity in biochemical experiments [42]. Many lower vertebrates (fishes and amphibians) and invertebrates (insects) have a Leu62Ile substitution at this position. To study the effect of this substitution on Pol ι activity and fidelity we tested yeast extracts producing the GST- Pol ι variant with substitution Leu62Ile (Pol ι^L62I^) in the misGvA assay. The pattern of bands was similar to the wild-type enzyme (right set of reactions of Fig. 3AB).

Because of the possibility of alternative splicing of the *POLI* transcript in humans and mice resulting in skipping exon 2, we produced the GST-tagged Pol ι variant with the deletion of exon 2 (Pol ι ^2exon-d^). Exon 2 consists of 42 amino acids and 29 residues are participating in the formation of the active site. This deletion should result in a significant change of the Pol ι biochemical properties or a complete loss of the enzymatic activity. Indeed, no activity has been detected (the right set of reactions in Fig. 3AB).

### misGvA activity of purified variants of Pol ι

**Figure 4:**
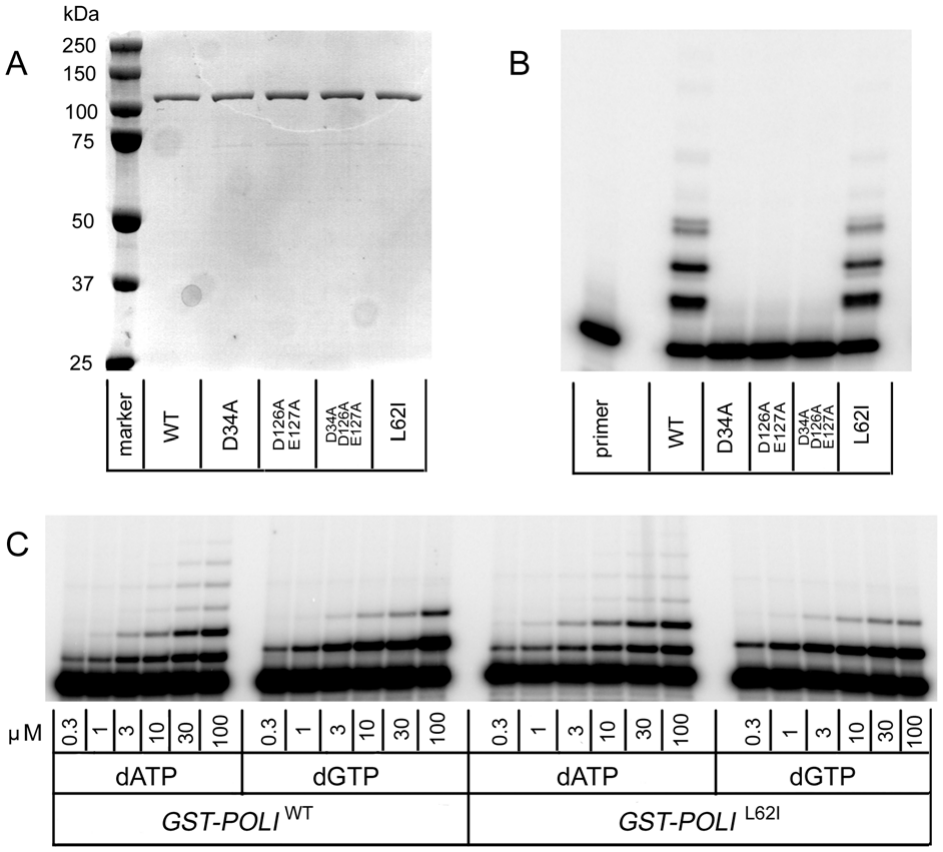
Activity of pure GST-tagged Pol ι variants. A. Purification of GST-Pol ι and its variants by affinity chromatography: the photograph of a Coomassie brilliant blue stained gel is shown. Equal volumes (15 µl) of each fraction with wild-type GST-Pol ι, GST-Pol ι^D34A^, GST-Pol ι^D126A/E127A^, GST-Pol ι^D34A/126A/E127A^, and GST-Pol ι^L62I^ eluted from the glutathione-sepharose column were analyzed on 8% SDS-PAGE. B. The comparative DNA-polymerase assay with purified GST-Pol ι and its variants. The ability of enzymes to extend a P^32^-labeled 17-mer primer annealed to template 1 was assayed in the presence of 100 µM of all four dNTPs and 0.15 mM Mn^2+^ ions, at 37°C for 5 min. C. Kinetic analysis of dATP and dGTP incorporation by purified wild-type GST–Pol ι and GST–Pol ι ^L62I^ variant. Primer extension reaction was carried out in the presence of 0.15 mM Mn^2+^ divalent metal ions and 1 nM of GST-Pol ι or its catalytically compromised variant at 37°C for 2.5 min. To quantify the incorporation of dATP and dGTP opposite template T we varied each dNTP concentration from 0.3 to 100 µM. Kinetic parameters determined from these experiments were: Wild-type: dATP: K_m_ = 3.5±1 µM, V_max_ = 9.8±0.8 (% incorporation/min), dGTP: K_m_ = 0.57±0.08 µM, V_max_ = 14.9±0.3 (% incorporation/min), f_inc_ for dGTP = 5.3; and L62I: dATP: K_m_ = 4.0<0.9 µM, V_max_ = 11.4±0.8 (% incorporation/min), dGTP: K_m_ = 0.54±0.09 µM, V_max_ = 17.2±0.3 (% incorporation/min), f_inc_ for dGTP = 6.1.

We purified Pol ι variants by affinity chromatography (Fig. 4A). The activity profile was qualitatively similar to the data obtained with extracts (Fig. 4B). Enzymes with a single D34A, double D126A/E127A, and triple D34A/D126A/E127A amino acid substitutions were completely inactive. The activity of the enzyme with the L62I change was similar to the wild-type enzyme. Quantitative kinetics analysis with the L62I mutant confirmed these observations (Fig. 4C and kinetic constants in the legend).

## Discussion

Mammalian Pol ι is a unique Pol with an unusual combination of properties, such as stimulation by Mn^2+^ ions, bypass of certain DNA lesions and the lowest fidelity among eukaryotic DNA polymerases. The exact biological role of Pol ι in humans and other organisms is still unclear [43].

Regulation of the eukaryotic Y-family DNA-polymerases is complex and depends on protein modifications, the formation of multi-enzyme complexes and protein–protein interactions [44]. The investigation of the DNA polymerases activity in nuclear and cellular extracts [45] provides additional important information to the studies of DNA Pols gene expression at the mRNA level and the activity of the purified enzymes *in vitro.* The knowledge of levels of the Y-family Pols gene expression in human cancer cells is important [20], but the information on the Pol activity is desirable for better understanding of the disease etiology and prognosis. We therefore introduced a method of detection of Pol ι biochemical activity directly in animal cell extracts [32]. Rigorous proof that the simple methodology specifically detects the activity of Pol ι was lacking. Mammalian cells possess numerous DNA polymerases and some of them, e.g. Pol η, have low fidelity. To provide the proof of the “misGvA” method and apply it to the different variants of Pol ι, we produced them in bakers yeast. Yeast does not have a *POLI* ortholog. We have found that the “misGvA” activity is absent in cell extracts containing an empty vector but it is robust in the strain expressing wild-type *POLI*. We also produced human Pol η, and have shown that these extracts generate a different pattern of bands compared to the bands of yeast extracts producing Pol ι. The misGvA technology was shown to be very efficient for the functional analysis of several variants of human Pol ι produced in yeast. We have found by misGvA method that the Pol ι variant lacking exon 2 is completely inactive. Amino acids D34, D126, E127 (Fig. 1B) are critical for the activity of the enzyme and a change of L62I has a little if any effect on the properties of Pol ι. Because the variant lacking exon 2 deletes a stretch of amino acids including D34 (Fig. 1A), its inactivity is inevitable. The Leu62 was shown to be functionally important for human Pol ι [42]. We speculated previously that polymorphic substitution I62L has evolutionary significance [34]. The present results do not support this hypothesis but do not rule it out completely, because the change was done in human enzyme. Assays of Pol ι activity from lower vertebrates with Ile at a position equivalent to Leu62 could be interesting in this respect.

The “misGvA” activity of Pol ι in the presence of Mn^2+^ is not detected in extracts of the 129/J strain of mice with a stop-codon mutation in exon 2 of the *Poli* gene (Makarova, unpublished observation). Weak Pol ι-like activity with Mg^2+^ was seen though the brain of the 129/J strain of mice [32]. We proposed that the mechanism of this suppression is alternative splicing of exon 2 with the elimination of the stop codon and restoration of ORF. The results obtained in the present study allowed us to dismiss the “exon 2 splicing” hypothesis of the mechanism of suppression in brains of 129/J mice. There could, however, be other explanations of the apparent misGvA activity despite the nonsense mutation. It is possible that some nonsense-suppression mechanism operates in the brain. It is also possible that other inaccurate DNA Pols are more active in the presence of Mg^2+^ ions than in Mn^2+^ cations. This misGvA activity may be the result of the transient misalignment or error-prone behavior of Pols, because the substrate used in the aforementioned studies, allows the detection of some misGvA activity due to transient misalignment.

In our work, we improved the misGvA technique for specific detection of Pol ι replacing Mg^2+^ by Mn^2+^ ions in the reaction mixture, adapted the misGvA method to yeast extracts producing human DNA polymerases and demonstrated that this is a simple approach to detect the presence of Pol ι activity without time and cost-consuming biochemical manipulations. The purification of the enzyme takes hours and requires several liters of yeast culture. Our method with extracts is robust and the test could be performed only with 10–20 ml of culture, which is easy to make fresh for each experiment.

We propose that the “misGvA” method can be applied for detection and fast screening of activity Pol ι and study of catalytic properties of its variants in human cancers cell extracts as well as cells harboring Pol ι polymorphisms. The modifications of the method with extracts tailored for the detection of different kinds of DNA synthesis errors could be applied also as a simple approach to monitor replication fidelity in cell extracts.

The DNA synthesis in yeast extracts producing human Pol ι and Pol η was characterized by very low fidelity (high frequency of misincorporations). This did not directly translate into genomic instability *in vivo.* We did not observe elevated mutation rates in the yeast strains carrying plasmids expressing genes encoding for human Pol ι and Pol η (Table 1). The results are consistent with the absence of a direct mutator effect in yeast of overproduced yeast Pol η [46] and only mild effects on the protection from DNA damaging agents of repair-defective yeast producing human Pol ι [47]. Complex interactions within live cells tightly regulate the access of error-prone Pols to chromatin. Systems of ectopic co-expression of Y-family Pol genes with polymerase accessory factors could potentially help to reveal these regulators.

## Materials and Methods

### Vectors and yeast strains for the production of Pol η, wild-type and variant forms of Pol ι

Human *POLI* and *POLH* genes were chemically synthesized and optimized to yeast codon usage and then cloned to yeast expression vector pESC-URA (Stratagene) at the *Xho*I-*Nhe*I and *Bgl*II-*Sac*I sites, respectively, by the McLab Company (San Francisco, CA). Sequences of constructs, named *hPOLISc* and *hPOLHSc*, are available upon request. The *hPOLISc* was under *GAL1* yeast promoter and encodes for Pol ι with the c-myc-tag at the N-terminus. A similar construct was made with a similar expression vector but with *TRP1* marker, pESC-TRP. The *hPOLHSc* was under yeast *GAL10* promoter and encodes for Pol η with the FLAG-tag at the N-terminus. The production of Pol ι from these constructs was confirmed by Western blot.

To create the yeast expression vector encoding for Pol ι fused with GST (glutatione-S-transferase) at the N-terminus, the chemically synthesized *hPOLISc* gene was amplified by PCR and cloned in frame with GST into *Cla*I-*Sal*I sites of the pRS424-GAL-GST-TRP plasmid [48]; [49]. The expression of the *hPOLISc* gene was under the chimeric *GAL1-GAL10* yeast promoter.

Mutant forms of the *GST-hPOLISc* gene were constructed by using the Quick Change mutagenesis kit (Stratagene). To make deletions, we PCR-amplified the part of the plasmid, which should be left after deletion, DpnI treated the sample and ligated the resulting products after end-phosphorylation. The following pairs of primers were used for site-directed mutagenesis; the site of change is underlined.

For amino acid substitution D34A (Pol ι^D34A^ and Pol ι^D34A/D126A/E127A^ variants):

SM-D34-1 5′gtagaaacagtccaaggcaacgtgaacgataactc 3′and

SM-D34A-2 5′gagttatcgttcacgttgccttggactgtttctac 3′.

For amino acid substitution of adjacent residues D126A and E127A (Pol ι^D126A/E127A^ and Pol ι^D34A/D126A/E127A^ variants): 

SM-D126A-E127A-1 – 5′-gttgaaagattgggtttcgccgcaaacttcgttgacttgac-3′ and

SM-D126A-E127A-2 – 5′-gtcaagtcaacgaagtttgcggcgaaacccaatctttcaac-3′.

For L62I amino acid substitution (Pol ι^L62I^):

SM-L62I-1 –5′-gtgttcaacaaaagtacatcgttgttacctgtaactac-3′ and

SM-L62I-2 –5′-gtagttacaggtaacaacgatgtacttttgttgaacac-3′.

For exon 2 deletion variant (Pol ι^2exo-d^):

DM-2exo-1 –5′-cttgagaagaagcagcagcac-3′ and

DM-2exo-2 – 5′-gtgttcaacaaaagtacttg-3′.

For 421-715 amino acid deletion variant (Pol ι^421-715-d^):

DM-421-715-1 – 5′-cttagcggtgttcaaagcc-3′ and

DM-421-715-2 – 5′-taagtcgacctcgaggg-3′.

The production of Pol η, wild-type and catalytically compromised variants of Pol ι was performed in the *S. cerevisiae* protease-deficient strain BJ 2168 as described [49]; [50]. Mutation rates in yeast expressing *hPOLISc* or *hPOLHSc* encoding c-myc-Pol ι and FLAG-Pol η were measured in independent 5 ml cultures of transformants of strain Δl(-2)l-7B-YUNI300 [51] grown for two days in synthetic complete medium containing 2% galactose instead of glucose and selective for plasmid marker as described earlier [46]. As a positive control we have used transformants with pESC-LEU plasmid expressing the *pmCDA1* gene, encoding for highly mutagenic cytidine deaminase from lamprey [40].

### DNA templates

The three complementary oligonucleotides (one primer and two templates) that hybridize to form a duplex with a protruding 5′ end were used to create two substrates for DNA-polymerase reactions. Universal primer – 5′-GGAAGAAGAAGTATGTT-3′ and template **1** – 5′-CCTTCTTCATTCTAACATACTTCTTCTTCC-3′ were the same as in the pioneer work of [4]. (The first template T nucleotide at position 18 and next C at 19 are underlined). The template **2** – 5′-CCTTCTTCATTGTAACATACTTCTTCTTCC-3′ oligonucleotide is similar to template **1**, but contains G instead of C at position 19 of the 30-mer template (underlined). For this template, primer misincorporation of G opposite T can not occur by “transient misalignment” mechanism. All oligonucleotides were synthesized in the Eppley Institute Molecular Biology Core Facility and were PAAG-purified prior to use.

The primer was 5′-labeled with [γ-^32P^]ATP (MP Biochemicals #35020, end-labeling grade, >7,000 Ci/mmol) using T4 polynucleotide kinase and purified on Sephadex G25 Microspin columns (Amersham Biosciences).

Radio-labeled DNA substrates were prepared by annealing the 5′-P^32^-labeled 17-mer universal primer to the unlabeled templates at a molar ratio of 1∶2.

### “misGvA” activity in cell extracts

We have used the misGvA method of detection of Pol ι in crude extracts of cells described by [31]; [32] with modifications. The cell extracts were prepared from fresh unfrozen samples.

The extracts of yeast strain BJ2168 transformants with either vector alone or producing Pol η, Pol ι, Pol ι mutant forms were prepared by the disruption of cells with glass beads in cell lysis buffer (10% sucrose, 300 mM NaCl, 50 mM Tris-HCl (pH 7.5), 10 mM β-mercaptoethanol, 1 mM PMSF, and 2x Complete EDTA-free protease inhibitor cocktail (Roche). The buffer was added in 1 ml per 1 g of cells ratio to 1.5 ml Eppendorf tubes, which we vigorously shook five times for one minute in a Disruptor Genie (Scientific Industries Inc, Bohemia, NY, U.S.A.). The lysates of cells were clarified by centrifugation at 10,000 g for 10 min at 0°C. The protein concentration was measured by Bradford assay dye reagent concentrate (Bio-Rad laboratories) and adjusted to 10 mg/ml. The extracts were used as enzyme preparation for DNA-polymerase reaction with P^32^-labelled oligonucleotide substrate. The standard reaction was in a total volume of 12.5 µl and included 2.5 µl of fresh cells extract, 50 mM Tris-HCl (pH 8.0), 0.15 mM MnCl_2_ or 0.25 mM MgCl_2_, 30 nM of the substrate and 1 mM of each ultra pure dNTPs from Applied Biosystems. A variation of five different conditions is specified in detail in the legend to the figures. The reaction was carried out at 37°C for 8–15 min and stopped by cooling on ice and the addition of an equal amount of loading buffer (95% formamide, 10 mM EDTA, 0.1% bromophenol blue and 0.1% xylene cyanol). Three µl of the reaction mixture was run on 18% polyacrylamide/7 M urea 45 cm long gels (ratio of AA∶BAA = 37∶1). After electrophoresis, the gel was fixed in 10% glacial acetic acid and 30% ethanol, dried on a vacuum gel dryer with heating (80°C) and bands were visualized using Storage Phosphor Screen in Typhoon 9700 (GE, U.S.A.).

### Western blot analysis

To estimate the production of Pol η, Pol ι and Pol ι mutant forms, the same crude yeast cell extracts prepared for misGvA assay (or prepared similarly) were used for Western blot analysis. Samples containing 40 µg of total protein were subjected to electrophoresis in SDS-8% PAGE gels. Proteins were electro-transferred to an PVDF membrane (Millipore) and subsequently probed with a 1∶500 dilution of anti-GST polyclonal rabbit antibody (ab9085, Abcam) to detect GST-Pol ι and GST-Pol ι mutant forms, with a 1∶200 dilution of anti-myc monoclonal mouse antibody (# M4439, Sigma) to detect c-myc-Pol ι and with a 1∶1000 dilution of anti-FLAG mouse antibody (Sigma, # F3165) to detect FLAG-Pol η, respectively. Proteins were subsequently visualized using the Western Breeze Chromogenic Anti-Mouse and Anti-Rabbit Kits (Invitrogen).

### Purification of wild-type and mutant forms of GST-tagged Pol ι

The 100 g of yeast cells “popcorn” [50] were disrupted in Freezer Mill 6870 (SPEX Sample Prep LLC, NJ, U.S.A.) in liquid nitrogen by seven disruption cycles (2 min each at 15 pulses/sec with 2 min cooling intervals). Frozen extract powder was added to 350 ml of Binding buffer (120 mM NaCl, 15 mM K_2_HPO_4,_ 30 mM Tris HCl (pH 7.4), 5% glycerol, 1 mM EDTA, 10 mM β-ME, 1 mM PMSF, and 1x protease inhibitor cocktail (Roche)). All of the following procedures were at 0–4°C. When all powder was dissolved, the resulting mixture was centrifuged twice at 5300 rpm in a Beckman Avanti J20XP centrifuge in JS 5.3 rotor for 20 min. The cleared supernatant was gently agitated with 4 ml of glutathione-sepharose resin (GE Healthcare) for 1.5 hours.

The beads were subsequently packed into a disposable BioRad column, washed with eight column volumes of Binding buffer and 12 column volumes of Wash buffer (600 mM NaCl, 15 mM K_2_HPO_4,_ 30 mM Tris HCl (pH = 7.4), 5% glycerol, 10 mM β-ME). The GST–Pol ι fusion proteins were eluted from the column by stepwise washes with a four column volume of Elution buffer (30 mM of reduced glutathione, 100 mM NaCl, 15 mM K_2_HPO_4,_ 30 mM TrisHCl (pH = 7.4), 5% glycerol, 10 mM β-ME) and stored at −80°C. All variants except GST fused variant with the deletion of exon 2, which did not bind to the glutathione-sepharose resin, were approximately 95% pure after these procedures (Fig. 4A of the Results).

### Primer extension reaction and kinetic analysis of replication products for purified Pol ι and its mutant forms

Standard primer extension reaction of 12.5 µl was carried out with 0.15 mM MnCl_2_, 20 nM of substrate, 0.1 mM of each ultra pure dNTP, 2.5% glycerol, 0.1 mg/ml bovine serum albumin, and 2.6 nM of GST-Pol ι or its catalytically compromised variant. After incubation at 37°C for 5 min, reactions were terminated and processed as described previously. To quantify the incorporation of dATP and dGTP opposite template T by steady state kinetics analysis [52] we varied each dNTP concentration from 0.3 to 100 µM in 2.5-min reactions. The concentration of GST-Pol ι and GST-Pol ι^L62I^ variant for kinetic analysis was 1 nM.

Results were analyzed using Image Quant 5.2 software (GE). Saturation plots of velocity as a function of dNTP concentrations were calculated as a percentage of the primer extension per min. Calculations of V_max_ and K_m_ were performed using nonlinear least square fits with the GraFit program (Erithacus Software, UK).
